# Influence of Anodizing Conditions on Biotribological and Micromechanical Properties of Ti–13Zr–13Nb Alloy

**DOI:** 10.3390/ma16031237

**Published:** 2023-01-31

**Authors:** Agnieszka Stróż, Joanna Maszybrocka, Tomasz Goryczka, Karolina Dudek, Patrycja Osak, Bożena Łosiewicz

**Affiliations:** 1 Institute of Materials Engineering, Faculty of Science and Technology, University of Silesia in Katowice, 75 Pułku Piechoty 1A, 41-500 Chorzów, Poland; 2 Refractory Materials Center, Institute of Ceramics and Building Materials, Łukasiewicz Research Network, Toszecka 99, 44-100 Gliwice, Poland

**Keywords:** anodizing, biomaterials, biotribology, oxide nanotubes, Ti–13Zr–13Nb alloy

## Abstract

The biomedical Ti–13Zr–13Nb bi-phase (α + β) alloy for long-term applications in implantology has recently been developed. The porous oxide nanotubes’ (ONTs) layers of various geometries and lengths on the Ti–13Zr–13Nb alloy surface can be produced by anodizing to improve osseointegration. This work was aimed at how anodizing conditions determinatine the micromechanical and biotribological properties of the Ti–13Zr–13Nb alloy. First-generation (1G), second-generation (2G), and third-generation (3G) ONT layers were produced on the Ti–13Zr–13Nb alloy surface by anodizing. The microstructure was characterized using SEM. Micromechanical properties were investigated by the Vickers microhardness test under variable loads. Biotribological properties were examined in Ringer’s solution in a reciprocating motion in the ball-on-flat system. The 2D roughness profiles method was used to assess the wear tracks of the tested materials. Wear scars’ analysis of the ZrO_2_ ball was performed using optical microscopy. It was found that the composition of the electrolyte with the presence of fluoride ions was an essential factor influencing the micromechanical and biotribological properties of the obtained ONT layers. The three-body abrasion wear mechanism was proposed to explain the biotribological wear in Ringer’s solution for the Ti–13Zr–13Nb alloy before and after anodizing.

## 1. Introduction

Medical implants, which are a substitute for bone parts in the human body, are accompanied by the action of friction, which includes a set of phenomena occurring in the contact area of two bodies moving relative to each other, as a result of which resistance to movement arises [1,2,3,4,5]. As a consequence, the continuity of the passive layer or protective coating is broken, and then the native structure of the alloy changes. Thus, the protection against the corrosive environment is lost [6,7,8,9,10,11,12,13,14]. Biotribology deals with the search for minimizing the effects of friction, which would involve reducing the wear of the surfaces involved in the friction of the elements and reducing the energy accompanying the process [15,16,17,18,19,20,21,22,23].

Currently, four basic groups of metallic biomaterials can be distinguished, among which the most widely used are austenitic steels [4]. The second group consists of shape memory alloys belonging to smart materials [4,14]. For long-term implants, the service life of which should not exceed 15 years, cobalt alloys are applied [4]. Titanium and titanium alloys are most often used due to their best biocompatibility, and their service life may exceed 20 years [1,2,3,4,5,6,7,8,9,10,11,12,13,15,16,17,19,21]. The market of long-term implants is dominated by a commercially pure titanium alloy (Cp Ti) and a bi-phase (α + β) Ti–6Al–4V alloy known also as Grade 5 Titanium or Ti 6–4, which has excellent strength, a low modulus of elasticity, high corrosion resistance, good weldability, and is heat treatable [19,23]. Al and V alloy additions increase the hardness of titanium and improve its physical and mechanical properties. However, the Ti–6Al–4V alloy shows unfavorable tribological properties, and its Young’s modulus (~110 GPa) is higher than that of bone (~10–64 GPa). Aluminum has well-documented toxicity in the serum or urine of patients; moreover, it has a causal relationship with neurotoxicity and senile dementia of the Alzheimer type. In addition, vanadium is thermodynamically unstable in conditions corresponding to the tissue environment and is considered a toxic alloying additive, similarly to vanadium oxide (V_2_O_5_), which has relatively good solubility and high toxicity in living organisms. Therefore, growing doubts about the cytotoxicity of the Ti–6Al–4V alloy have prompted investigations into the development of modern vanadium-free Ti alloys containing biocompatible elements such as Mo, Nb, Zr, and Ta, which are able to stabilize the β structure in titanium [10,24,25,26].

One of the newest classes of biomedical alloys which excludes harmful elements in the form of Al and V from the composition is the Ti–13Zr–13Nb alloy developed by Davidson and Kovacs [24]. The Ti–13Zr–13Nb alloy combines a low modulus with high strength and excellent hot and cold serviceability. Research on this alloy has shown that its mechanical properties can be controlled to a large extent by hot working, heat treating, and cold working. The Ti–13Nb–13Zr alloy also shows high corrosion resistance in a physiological environment, which is one of the most important factors that has a decisive impact on the use of a given biomaterial for implants [7,11,12,24,27]. Like titanium, niobium is an element with high corrosion resistance, which is due to its susceptibility to being covered with a self-passive Nb_2_O_5_ oxide layer [28]. Zirconium has similar physicochemical properties to those of titanium and can be processed by similar methods. In some strength parameters, this element even surpasses titanium [23]. Zr is not ferromagnetic, which allows the use of implants made of the Ti–13Zr–13Nb alloy in patients undergoing nuclear magnetic resonance imaging. The Ti–13Nb–13Zr alloy is characterized by favorable mechanical properties for implant applications and a low Young’s modulus. The alloys used for the production of biomaterials should have mechanical properties close to those they replace, therefore, the modulus of elasticity of Ti–13Zr–13Nb may vary between 41 and 83 GPa.

The natural and uneven oxide layer present on titanium and its alloys does not sufficiently protect the implant in the environment of body fluids, therefore the surfaces of these materials are often subjected to modification [6,7,8,11,12,13,14,29,30]. One of the currently most popular electrochemical methods of modifying the oxide film to form self-assembled nanotubular oxide structures is anodizing, which allows the production of oxide layers in the form of a matrix of ordered, vertically arranged oxide nanotubes (ONTs) [11,12,13,17,18,19,28,29,31,32,33,34,35,36,37,38,39,40]. The ONTs exhibit numerous unique properties compared to ultrathin oxide films formed spontaneously. Increasing the surface roughness at the nanoscale by producing ONTs contributes to a better adhesion tendency of bone-forming cells. The porous surface of the ONT layers has a beneficial effect on the osseointegration process and ensures faster tissue growth and stronger bonding of the bone with the implant due to the chemical and morphological similarity of the nanotube oxide layer to the structure of bone tissue. The type of electrolyte in which the anodizing process is carried out has the greatest impact on the microstructure and properties of the ONTs obtained on titanium oxide and its alloys [11,12,13,17,18,19,28,29,31,32,33,34,35,36,37,38,39,40]. The prospect of using ONTs dominated the efforts of researchers at the expense of understanding the mechanical properties of the nanotubular oxide layer [21,23,36,41,42,43]. Most biocompatibility studies of ONTs focus on their use in dentistry, orthopedics, and cardiovascular surgery due to their high affinity for bone cell adhesion and differentiation, hydroxyapatite formation, and outstanding biochemical inertia [1,2,3,4,5]. ONT layers on the surface of titanium and its alloys are considered promising bionanomaterials for controlled drug delivery systems to suppress local inflammation after the implantation process [29]. The possibility of selecting anodizing parameters when obtaining ONTs with desired, predetermined morphological features can be used in practical applications in personalized medicine. ONTs used as intelligent drug carriers enable the delivery of poorly water-soluble drugs and prevent the first-pass effect through the liver. ONTs can be produced on a variety of substrates, including three-dimensional, nonplanar, and curved surfaces such as, for example, thin, long surgical wires and bone fixation needles, allowing ONTs to be clinically used on the surface of implants or surgical supports in orthopedics [31,32,33,34,35,36,37,38,39,40]. However, despite numerous studies on ONTs, relatively little is known about their impact on biotribological wear of modern titanium alloys in a biological environment due to the dominance of research in dry sliding conditions [17,19].

This work is a continuation of our interest in the surface functionalization of the biomedical Ti–13Zr–13Nb alloy through the production of ONTs of the first, second, and third generation. The main purpose of the undertaken research is to determine for the first time the effect of anodizing conditions on biotribological wear in Ringer’s solution and micromechanical properties of the Ti–13Zr–13Nb alloy for the development of long-term implants. This work brings new insights into the relationship between the anodizing conditions and biotribological wear of the Ti–13Zr–13Nb alloy under wet sliding conditions.

## 2. Materials and Methods

### 2.1. Substrate Surface Treatment

The substrate material was a rod with a diameter of 9 mm and a length of 1 m made of a bi-phase Ti–13Zr–13Nb alloy composed of a mixture of α and β phases (BIMO TECH, Wrocław, Poland) with a chemical composition in wt% according to the standard ASTM F1713-08(2021)e1 [44]. One side of the cut samples in the shape of disks with a thickness of 5 mm was subjected to wet grinding and polishing using a metallographic grinding and polishing machine Metkon Forcipol 102 (Metkon Instruments Inc., Bursa, Turkey), equipped with an automatic header. The samples were embedded into conductive PolyFast resin (Struers, Cleveland, OH, USA) using an ATM Opal 400 hot mounting press (Spectrographic Ltd., Guiseley, Leeds, UK) at 180 °C for 10 min. A mirror-like surface of the substrate was obtained on a wheel used for grinding at 250 rpm with water-based silicon carbide abrasive papers of P600 to P2500 gradations (Buehler Ltd., Lake Bluff, IL, USA). Diamond suspensions with 6 to 1 μm grain size (Buehler, Waukegan, IL, USA) were used for further polishing. Polishing was finished using a polishing cloth (Buehler, Waukegan, IL, USA) and a colloidal SiO_2_ suspension with a grain size of 0.04 μm (Struers, Cleveland, OH, USA).

The polished samples were cleaned for 20 min in an ultrasonic cleaner USC-TH (VWR International, Radnor, PA, USA) with acetone (Avantor Performance Materials Poland S.A., Gliwice, Poland) and then in ultrapure water with resistivity of 18.2 MΩ cm (Milli-Q Advantage A10 Water Purification System, Millipore SAS, Molsheim, France) with two Milli-Q water changes. As a result of high-frequency ultrasonic waves propagating in the liquid, vacuum bubbles were formed, under the influence of which rapid evaporation of the liquid and the formation of water vapor bubbles took place. During the implosion of the vacuum bubbles, a local increase in temperature and pressure was generated. The imploding bubbles, located at the contaminated surface, detached the pollutants.

### 2.2. Anodizing Conditions of Ti–13Zr–13Nb Alloy

Anodes made of the Ti–13Zr–13Nb alloy with a one-sided geometric surface area of 3.14 cm^2^ were prepared in accordance with the detailed information provided in our earlier work [29]. Immediately before anodizing, the prepared electrodes were immersed in 25% *v*/*v* HNO_3_ (Avantor Performance Materials Poland S.A., Gliwice, Poland) for 10 min at room temperature to remove oxides from the surface of the Ti–13Zr–13Nb alloy, and then sonicated in Milli-Q water for 20 min.

The ONTs on the Ti–13Zr–13Nb electrode surface were produced in a two-electrode system by one-step anodizing at room temperature under the conditions shown in Table 1.

Hydrofluoric acid (48 wt.% in H_2_O, ≥99.99% trace metals basis), ammonium sulfate (for molecular biology, ≥99.0%), ammonium fluoride (≥99.99% trace metals basis), and ethylene glycol (anhydrous, 99.8%) were supplied by Sigma-Aldrich (Saint Louis, MO, USA). A PWR800H high-current power supply (Kikusui Electronics Corporation, Yokohama, Japan) was used. The anodes with freshly prepared ONTs were placed in Milli-Q water, which was vigorously stirred for 5 min.

### 2.3. Physicochemical Characteristics of ONTs on Ti–13Zr–13Nb Alloy

The microstructure of the Ti–13Zr–13Nb alloy before and after anodizing was studied using a TESCAN Mira 3 LMU scanning electron microscope (SEM, TESCAN ORSAY HOLDING, Brno-Kohoutovice, Czech Republic). The secondary electrons (SE) were used for imaging that were generated by the incoming electron beam as they entered the surface. The use of SE allowed to obtain a high-resolution signal with a resolution that was only limited by the electron beam diameter. The SEM examinations were performed on the samples covered by an ultrathin layer of chromium deposited using a Quorum Q150T ES Sputter Coater (Quorum Technologies, East Sussex, UK).

### 2.4. Microhardness of ONTs on Ti–13Zr–13Nb Alloy

The microhardness of the Ti–13Zr–13Nb alloy before and after anodizing was determined using the Vickers method by means of a Wilson^®^–WolpertTM Microindentation Tester 401MVD (Wilson Instruments, LLC, Carthage, TX, USA). A hardness scale of HV = 0.1 was used. A Vickers indenter was applied, which was a square-based pyramidal-shaped diamond indenter with face angles of 136° according to the ISO 6507-1 standard [45]. The Vickers microhardness measurements were performed with variable loads of 490, 980, 1560, and 4900 mN, respectively. Indentation diagonal lengths were between 0.020 and 1.400 mm. The Vickers hardness number was calculated based on Equation (1):

(1) $$\mu \mathrm{HV}=\frac{1854.4\times P}{d^{2}}$$

where P—force (gf) and d—mean diagonal length of the indentation (μm).

A direct method of checking and calibrating the microhardness tester, indenter, and diagonal length of the measuring system was used according to the ISO 6507-2:2018 standard [46].

### 2.5. Biotribology of ONTs on Ti–13Zr–13Nb Alloy in Ringer’s Solution

**Figure 1:**
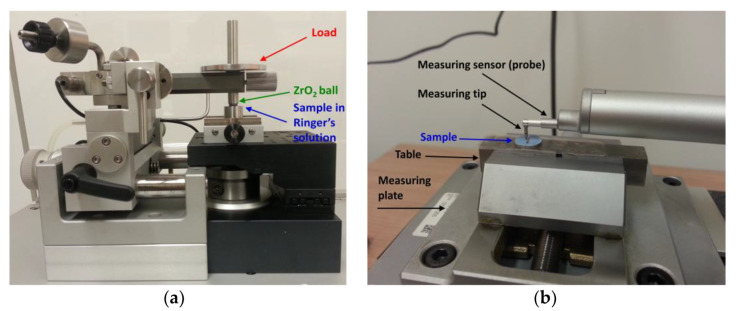
Setup for testing biotribological wear: (**a**) tribometer for measurements in the ball-on-flat system with a load of 1 N; (**b**) method of arranging the sample before testing on the contact profilograph.

Measurements of biotribological wear of the Ti–13Zr–13Nb alloy in the initial state and after anodizing were performed in a reciprocating motion in the ball-on-flat system using a tribometer (Anton Paar Polska, Warsaw, Poland) shown in Figure 1a. The counter-sample in the test was a ZrO_2_ ball with a diameter of 6 mm. The tests were carried out in Ringer’s solution with the chemical composition (g cm^−3^): 8.60—NaCl, 0.30—KCl, and 0.33—CaCl_2_·2H_2_O. To adjust the pH of the solution to 7.4(1), 4% NaOH and 1% C_3_H_6_O_3_ were used. Chemical reagents of recognized analytical grade (Avantor Performance Materials Poland S.A., Gliwice, Poland) and Milli-Q water were used to prepare the solution. The normal force (F_n_) in the friction node was 1 N. A sliding rate (r) of 2.5 cm s^−1^ and stroke length (l) of 4 mm were applied. The biotribological test consisted of 3749 cycles (back and forth = 1 cycle), which corresponded to a total friction distance (s) of 30 m.

The assessment of biotribological wear of the tested samples was carried out based on profilometric analysis of wear scars using the SURFTEST SJ-500 profilometer (Mitutoyo Corporation, Kanagawa, Japan) shown in Figure 1b.

The specific wear rate (Vv) in mm^3^ N^−1^ m^−1^ was determined according to Equation (2):

(2) $$V_{V}=\frac{A\cdot I}{F_{n}\cdot s}$$

where A—the wear scar area (mm^2^), l—the stroke length (mm), F_n_—the normal force (N), and s—the friction distance (m).

The biotribological wear analysis of the ZrO_2_ ball was carried out based on measurements of the diameter of the wear scar on the counter-sample using a BX51 optical microscope (Olympus, Shinjuku, Tokyo, Japan). The microstructure was analyzed, with particular emphasis on the surface of the wear scar of the tested samples, resulting from prior biotribological testing.

Five samples of each type were tested, and the values of the determined parameters are the average values with their standard deviations (SD).

## 3. Results and Discussion

### 3.1. Microstructure of ONTs on Ti–13Zr–13Nb Alloy

**Figure 2:**
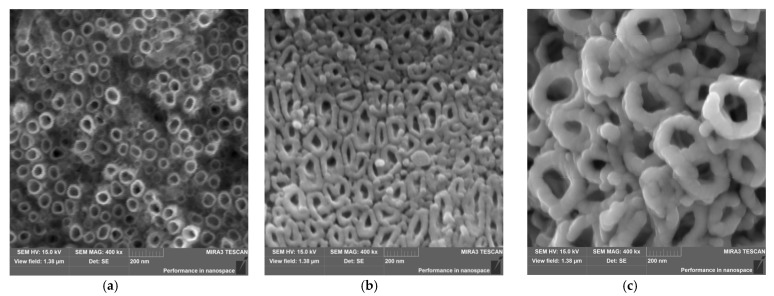
SEM image of the microstructure of ONT layer on the Ti–13Zr–13Nb alloy obtained by anodizing in: (**a**) 0.5% HF electrolyte (1G); (**b**) 1M (NH_4_)_2_SO_4_ + 2% NH_4_F electrolyte (2G); (**c**) 1M C_2_H_6_O_2_ + 4% NH_4_F electrolyte (3G).

Figure 2 shows SEM images of the microstructure of the self-assembled ONTs obtained on the surface of the Ti–13Zr–13Nb alloy by anodizing under different conditions (see Table 1). The detailed mechanism of the electrochemical formation of ONT layers on the surface of the Ti–13Zr–13Nb alloy in electrolytes containing fluoride ions that form water-soluble complexes with titanium, zirconium, and niobium was described in our earlier paper [33,35]. A strong influence of the type of electrolyte on the surface morphology of the obtained ONT layers could be observed. The on-top general view of the Ti–13Zr–13Nb alloy with the ONTs of 1G (Figure 2a), 2G (Figure 2b), and 3G (Figure 2c) revealed the presence of well-developed and evenly distributed oxide nanotubes with single and very smooth walls.

The 1G ONTs had the smallest inner diameter of 71(7) nm, an outer diameter of 87(10) nm, and nanotube length of 0.94(9) µm [32]. The wall thickness of the 1G ONTs was also the smallest among all the obtained generations of ONTs on the surface of the Ti–13Zr–13Nb alloy. The ONTs formed in the 0.5% HF electrolyte at 20 V for 120 min had a circular or elliptical cross-section (Figure 2a). The 2G ONTs were characterized by higher values of geometrical parameters of the produced nanotubes on the Ti–13Zr–13Nb alloy, which were determined based on SEM images from selected areas of the oxide surfaces (Figure 2b). The ONTs with an inner diameter of 61(11) nm and an external diameter of 103(16) nm were obtained in the electrolyte of 1M (NH_4_)_2_SO_4_ + 2% NH_4_F at 20 V for 120 min [34]. The length of vertically positioned 2G ONTs with a circular cross-section was 3.9(2) μm. The 2G ONTs were the most densely packed and tended to form clusters of oxide nanotubes of various lengths. The obtained results showed that using the organic electrolyte 1M C_2_H_6_O_2_ + 4% NH_4_F at 50 V for 80 min allowed obtaining an over three-fold increase in inner diameter and an about four-fold increase in outer diameter of 3G ONTs compared to that of the ONTs of 1G and 2G [31]. The ONTs of 3G with a circular cross-sectional shape had the thickest nanotube walls. The length of 3G ONTs increased more than 10-fold and two-fold compared to the length of 1G ONTs and 2G ONTs, respectively.

The obtained results indicate that the modification of the surface of the Ti–13Zr–13Nb alloy made it possible to obtain porous layers of ONTs with different morphological parameters and lengths. The surface morphology of all obtained generations of ONTs was very similar to the structure of trabecular bone, which will affect the ability to transfer load by implants made of the Ti–13Zr–13Nb alloy with an ONT layer applied. It was signaled in the literature that ONT layers on titanium and its alloys show the ability to accelerate osseointegration, leveling the inflammatory states and preventing the penetration of harmful corrosion products into the biological environment of the body [10,41,43]. The ONT layers can also be used as intelligent carriers of medicinal substances in drug delivery systems, especially for personalized medicine [29].

Long-term in vitro corrosion resistance studies of the Ti–13Zr–13Nb alloy in saline solution showed the influence of the electrode immersion time on the change in the thickness of the self-passive oxide layer [7]. In studies using the electrochemical impedance spectroscopy method, it was shown that the thickness of the self-passive oxide layer increased from 0.6 to 2.3 nm for 20 days of immersion. It should be emphasized that after 20 days of immersion tests, no pitting was observed on the surface of the Ti–13Zr–13Nb alloy. Comparative assessment of the determined corrosion resistance parameters showed that surface modification of the Ti–13Zr–13Nb alloy by anodizing increased its corrosion resistance in a saline solution for the 2G ONT layers [12] and slightly decreased for the 3G ONT layer [31] as compared to the non-anodized Ti–13Zr–13Nb alloy surface. It was shown that the in vitro corrosion resistance of ONT layers depended on their structure and morphological parameters. In the case of both the Ti–13Zr–13Nb alloy before and after the production of 2G and 3G ONT layers, no breakdown potential was revealed in potentiodynamic tests conducted up to 9.5 V, which indicates that the tested biomaterials had excellent resistance to pitting corrosion and can be promising biomaterials for long-term use in implantology.

### 3.2. Micromechanical Properties of ONTs on Ti–13Zr–13Nb Alloy

**Figure 3:**
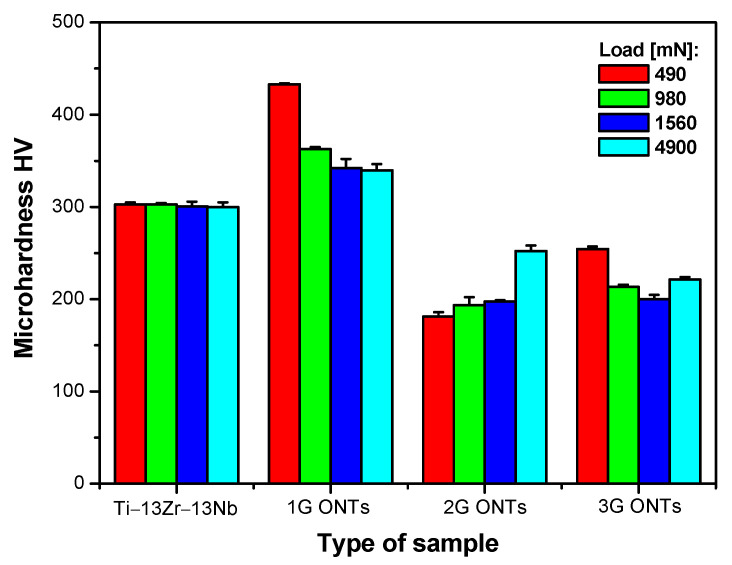
Vickers microhardness of the Ti–13Zr–13Nb alloy surface before and after anodizing with variable loads.

The assessment of the effect of anodizing conditions on the micromechanical properties of the Ti–13Zr–13Nb alloy was carried out based on the Vickers microhardness tests. Microhardness, which is the hardness of the material exposed to low applied loads, is particularly useful for assessing the structural integrity of the bone tissue on the surface of the porous layer of ONTs and the bone tissue that surrounds the implant in the process of osseointegration. For this reason, it is important to compare the micromechanical properties of the Ti–13Zr–13Nb alloy before and after anodizing. The quantitative assessment of the anodizing effect in the microscale consisted in determining the micromechanical properties of the Ti–13Zr–13Nb alloy before and after the formation of ONT layers in various conditions of electrochemical oxidation (Figure 3).

The results presented in Figure 3 indicate that Vickers microhardness of the Ti–13Zr–13Nb alloy changed as a result of anodizing. The value of Vickers microhardness for the non-anodized alloy determined under variable loads in the range from 490 to 4900 mN was constant within the error limits and amounted to 302(1). In the case of three generations of ONT layers, Vickers microhardness depended on the applied load, which resulted from the different lengths of the tested oxide nanotubes and their morphological parameters (Figure 2). Compared to the Ti–13Zr–13Nb alloy in the initial state, an increase in the microhardness was observed in the case of the 1G ONT layer, for which Vickers microhardness value decreased in the range from 433(1) to 340(7) with increasing load. Such micromechanical properties of the 1G ONT layer resulted from the smallest outer diameter of oxide nanotubes and their shortest length among all obtained generations of ONTs (Table 1). The 1G ONT layer had the largest number of individual nanotubes on the surface, and thus had the greatest load carrying capacity [18,19]. Vickers microhardness value dropped for the 2G and 3G ONT layers compared to that of the substrate surface and 1G ONT layer (Figure 3). The 2G ONT layers showed the smallest Vickers microhardness ranging from 181(5) to 252(6) with increasing load in the range of 490 to 4900 mN. The 3G ONT layers revealed a slightly higher Vickers microhardness than the 2G ONT layers did, which varied from 254(3) to 221(3) with increasing load. Larger outer diameters of the 2G and 3G ONTs and their greater length cause easier deformation and cracking of the obtained oxide nanotubes [17]. With the increase in the ONTs diameter on the Ti–13Zr–13Nb alloy, the number of oxide nanotubes carrying loads in the contact area of the tested surfaces with the diamond indenter decreased. On the other hand, ONTs are able to compensate for the large hardness defect of the biomedical Ti–13Zr–13Nb alloy used for the production of implants, eliminate implant–bone stress mismatch, and minimize “stress shielding” [35].

### 3.3. Biotribological Properties of ONTs on Ti–13Zr–13Nb Alloy

**Figure 4:**
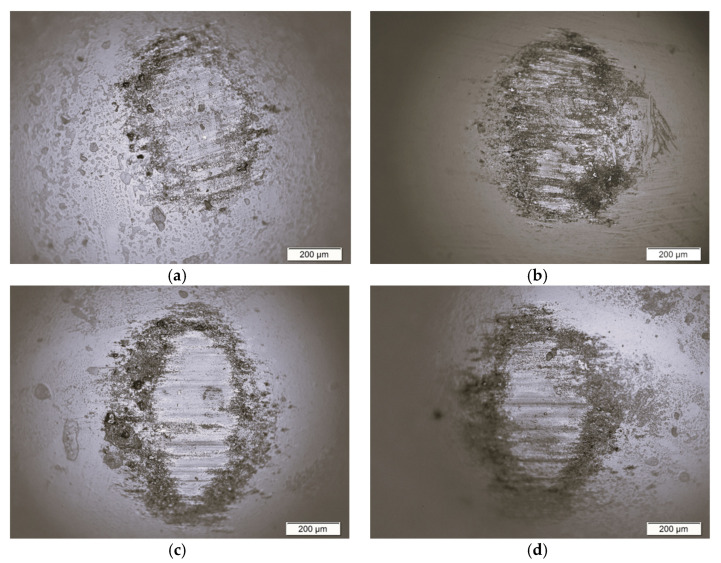
ZrO_2_ ball wear scar after the ball-on-flat biotribological test against (**a**) Ti–13Zr–13Nb substrate; (**b**) 1G ONT layer; (**c**) 2G ONT layer; (**d**) 3G ONT layer.

Biotribological wear resistance tests and friction coefficient measurements were carried out under sliding friction conditions in the presence of Ringer’s solution, which was a biological lubricating fluid. The Ti–13Zr–13Nb alloy in the initial state and with the 1G, 2G, and 3G ONT layers was subjected to biotribological tests in reciprocating motion in the ball-on-flat system, after which microscopic analysis of wear scars of the ZrO_2_ ball was performed (Figure 4).

On the microscopic images of counter-sample wear scars after the biotribological wear test, the direction of damage to the ball from top to bottom was observed for all tested materials. Residual abrasion of materials on the surface of the ZrO_2_ ball was also visible. The wear scar of the ZrO_2_ ball in combination with the surface of the Ti–13Zr–13Nb alloy was characterized by the smoothest surface with a small number of impurities transferred to the surface of the counter-sample compared to the tested ONT layers, which indicated the lowest biotribological wear of the alloy substrate. During the friction process of the 1G, 2G, and 3G ONT layers, numerous scratches appeared on the surface of the ZrO_2_ ball. Such an effect was caused by the presence of residual abrasion products (debris) in the form of particles of the ONTs, which were subject to abrasion during the friction process and constituted an additional factor damaging the surface, causing an increase in friction.

**Figure 5:**
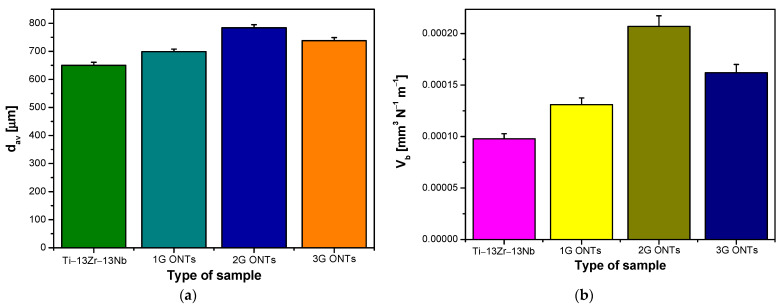
Parameters obtained after the biotribological wear test in the ball-on-flat system for the Ti–13Zr–13Nb alloy before and after anodizing: (**a**) ZrO_2_ ball wear scar (d_av_); (**b**) ZrO_2_ ball-specific wear (V_b_).

Based on microscopic observations, the average value of the ZrO_2_ ball wear scar diameter (d_av_) was determined, the values of which are shown in Figure 5a. The d_av_ value for the Ti–13Zr–13Nb alloy in the initial state was 650(11) µm. The d_av_ parameter assumed higher values in the presence of ONT layers produced on the alloy substrate, and thus indicated an increase in the specific wear of the counter-sample in the form of a ZrO_2_ ball (V_b_). Figure 5b shows a comparison of the V_b_ values determined for all tested materials. The ZrO_2_ ball wear scar for the 2G ONT layer had the largest width of d_av_ = 784(11) μm (Figure 5a) and showed the largest value of V_b_ equal to 2.07(10)·10^–4^ mm^3^ N^–1^ m^–1^ (Figure 5b), showing a more than two-fold increase in the biotribological wear of the counter-sample compared to V_b_ obtained for the ZrO_2_ ball-alloy Ti–13Zr–13Nb combination.

**Figure 6:**
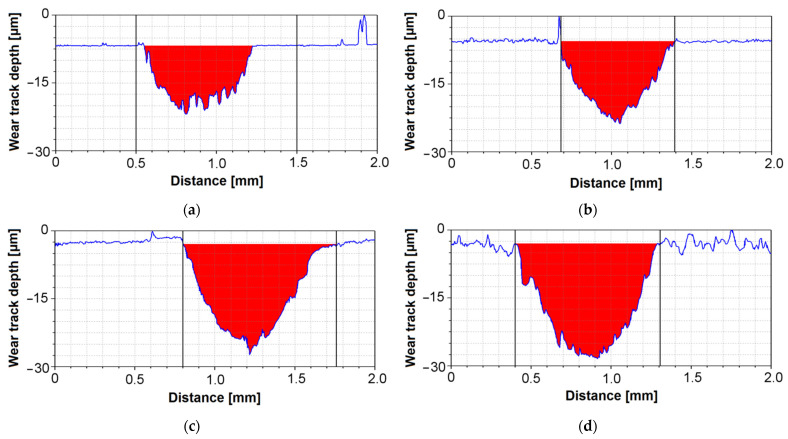
Cross-sectional profiles of wear tracks after the biotribological wear test in the ball-on-flat system for the Ti–13Zr–13Nb alloy before and after anodizing: (**a**) Ti–13Zr–13Nb alloy; (**b**) 1G ONT layer; (**c**) 2G ONT layer; (**d**) 3G ONT layer.

The results of profilometric tests of the wear track on the surface of the tested materials after the biotribological wear test in the ball-on-flat system are shown in Figure 6. Based on the obtained cross-sectional profiles of wear tracks for the Ti–13Zr–13Nb alloy before and after anodizing, it can be concluded that biotribological wear of the material surface depended on the anodizing conditions, while the surface of the Ti–13Zr–13Nb alloy in the initial state showed less material wear compared to anodized surfaces.

The width of the wear track for the surface of the Ti–13Zr–13Nb alloy determined based on the data in Figure 6 was 665(7) µm, and for electrochemically oxidized surfaces it increased, reaching the largest value of 827(8) µm for the 2G ONT layer. Larger values of the wear track width were observed for all the tested materials compared to the width of the ZrO_2_ ball wear scar (Figure 5a), which suggests a higher biotribological wear of the Ti–13Zr–13Nb alloy before and after anodizing compared to the ZrO_2_ ball used in a ball-on-flat combination.

**Figure 7:**
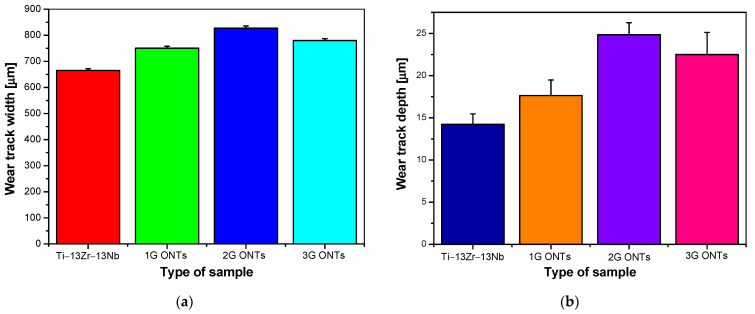
Parameters obtained after the biotribological wear test in the ball-on-flat system for the Ti–13Zr–13Nb alloy before and after anodizing: (**a**) wear track width; (**b**) wear track depth.

Figure 7b presents wear track depth obtained after the biotribological wear test for the Ti–13Zr–13Nb alloy before and after electrochemical oxidation. The smallest wear track depth was shown by the alloy substrate, the value of which was 14(1) µm. All generations of ONT layers showed greater wear track depth compared to that of the non-anodized substrate, with the greatest wear track depth value being 25(1) µm for the 2G ONT layer.

**Figure 8:**
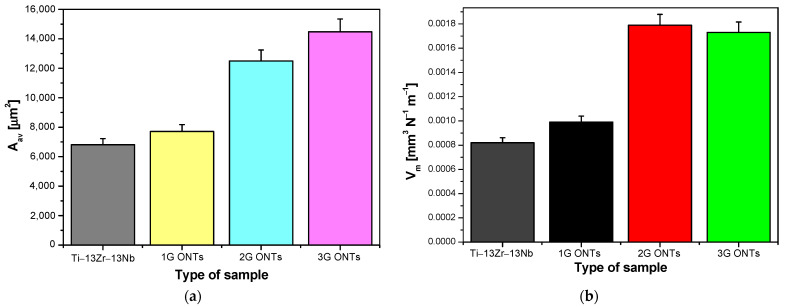
Parameters obtained after the tribological wear test in the ball-on-flat system for the Ti–13Zr–13Nb alloy before and after anodizing: (**a**) average wear surface area (A_av_); (**b**) average material volume consumption (V_m_).

Based on the d_av_ parameter (Figure 5a), which strongly depends on the type of the tested surface used in the friction node, the average wear surface area (A_av_) was determined after the biotribological test, which took the smallest value equal to 6814(64) µm^2^ for the Ti–13Zr–13Nb alloy in the initial state (Figure 8a). Along with the increase in the average outer diameter of ONTs and their length, an upward trend of A_av_ was observed. The A_v_ value determined for the non-anodized substrate was more than two times lower than the average wear surface area value for the 2G and 3G ONT layers.

Average material volume consumption (V_m_) took the lowest value of 8.20(4)·10^−4^ mm^3^ N^−1^ m^−1^ for the electrochemically unoxidized surface of the Ti–13Zr–13Nb alloy (Figure 8b). The obtained V_m_ value was twice as high as compared to the average material volume consumption for mechanically polished grade 4 titanium [15], 16 times higher than V_m_ for sandblasted grade 4 titanium [15], and 11 times higher than for sandblasted and steam-sterilized grade 4 titanium [16], subjected to biotribological wear test under comparable conditions in protein-free artificial saliva. The obtained results indicate that the value of V_m_ increased after the anodizing process of the Ti–13Zr–13Nb alloy in a solution of 0.5% HF, 1M (NH_4_)_2_SO_4_ + 2% NH_4_F, and 1M C_2_H_6_O_2_ + 4% NH_4_F. The highest value of Vm equal to 1.79(9)·10^−3^ mm^3^ N^−1^ m^−1^ was observed for the 2G ONT layer, which was very close to the V_m_ obtained for the 3G ONT layer within the limit of error (Figure 8b). Such a significant consumption of ONTs resulted from the porous structure of the tested layers. Oxide nanotubes are a kind of hollow tubes that carry load. During the friction process, the thin walls of 2G and 3G ONTs with a lower microhardness compared to that of the alloy substrate and 1G ONTs (Figure 3) break more easily, resulting in higher material consumption.

**Figure 9:**
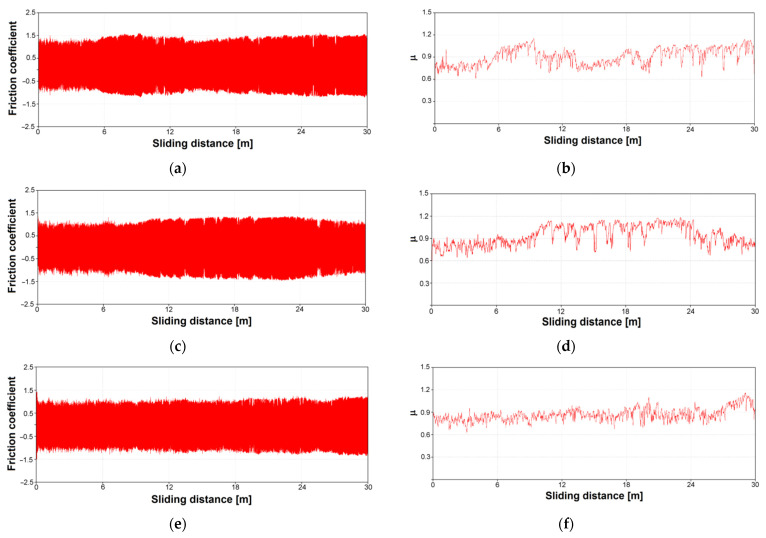

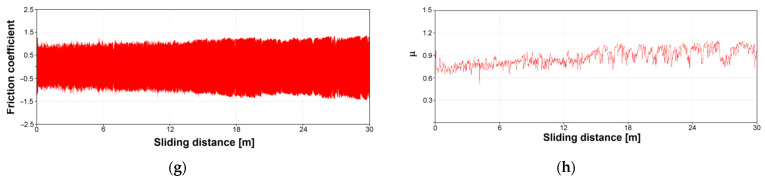
Friction coefficient (μ) as a function of the sliding distance in the ball-on-flat system for: (**a**,**b**) Ti–13Zr–13Nb alloy; (**c**,**d**) 1G ONT layer; (**e**,**f**) 2G ONT layer; (**g**,**h**) 3G ONT layer.

Figure 9 shows the course of the friction coefficient as a function of the sliding distance for the Ti–13Zr–13Nb alloy and its surface after anodizing. In the conducted biotribological tests, the coefficient of friction was a measure of the resistance of the tested materials in the process of friction against the counter-sample penetrating the material under study.

**Figure 10:**
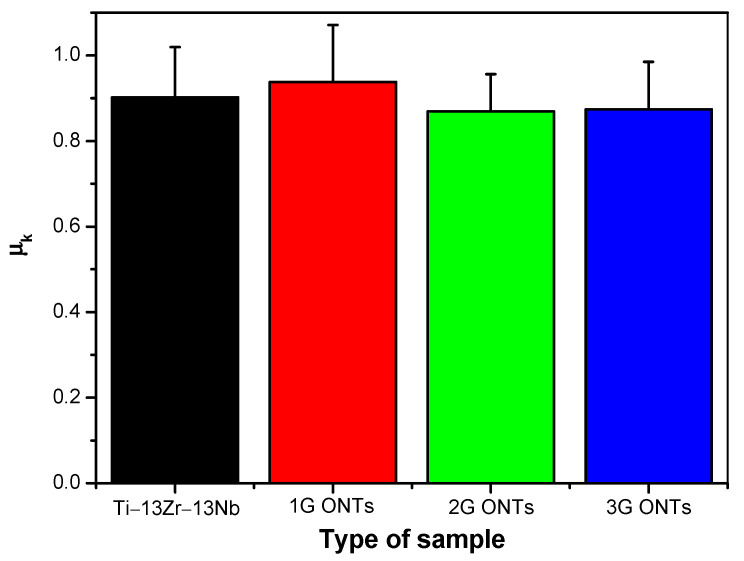
Kinetic coefficient of friction (µ_k_) obtained in the tribological wear test using the ball-on-flat system for the Ti–13Zr–13Nb alloy before and after anodizing.

In the graph of the coefficient of friction shown in Figure 9, the initial course of friction was attributed to the initial oxide layer, which was then systematically removed [20]. The initial lower friction coefficient values were mainly due to two factors. Firstly, the wear debris produced in the first stage filled the pores of the outer layer, which increased the contact area between the ZrO_2_ ball and the tested surface. The outermost layer of ONTs was easy to remove and was a carrier of particles that affected friction processes. Secondly, as the porous outer layer was gradually worn away, the counter-sample had increased contact with the denser inner layer. At the end of the friction process, particles formed in the friction process (wear debris) accumulated and the friction coefficient increased. Long nanotubes contributed to the accumulation of a large amount of worn material and thus a higher value of the friction coefficient [21,22]. On the graph of the friction coefficient for 2G ONTs, the smoothest course along the entire length of the sliding distance was visible, showing the easiest wear of the material (Figure 9e,f). Based on the friction coefficient, the kinetic coefficient of friction (µ_k_) was determined, which took the smallest value of 0.86(8) for the surface of the Ti–13Zr–13Nb alloy with a 2G ONT layer (Figure 10). A similar value of µ_k_ of 0.86(6) and 0.87(3) was determined for sandblasted [15] and sandblasted and sterilized [16] grade 4 titanium, respectively.

**Figure 11:**
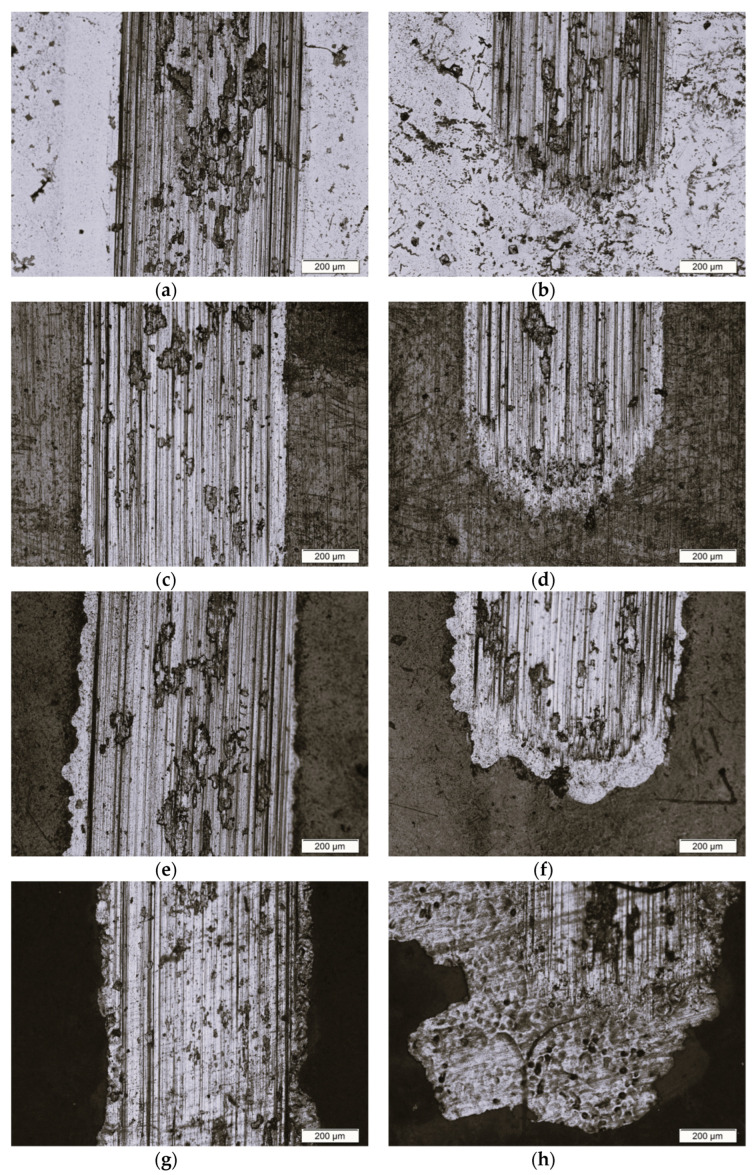
Microscopic image of wear track after the biotribological test in the ball-on-flat combination system for: (**a**,**b**) Ti–13Zr–13Nb alloy; (**c**,**d**) 1G ONT layer; (**e**,**f**) 2G ONT layer; (**g**,**h**) 3G ONT layer.

Figure 11 shows exemplary microscopic images of wear tracks of the tested materials after the biotribological test in Ringer’s solution performed in the center region of wear tracks (Figure 11a,c,e,g) and in the border of wear tracks and untested surfaces (Figure 11b,d,f,h).

Visible scratches and furrows in Figure 11 were the result of the movement of wear products along the working path of the counter-sample. The surface of the Ti–13Zr–13Nb alloy in the initial state had numerous material losses and pits filled with remnants of the abraded material and lubricant in the form of Ringer’s solution (Figure 11a,b). The surface of the 1G ONT layer (Figure 11c,d) and 2G ONT layer (Figure 11e,f) also showed numerous material losses. Accumulations of abraded material were visible in the contact area of the counter-sample with the track of abrasion. The surface with 3G ONTs showed delamination of the oxide layer (Figure 11g,h). In addition, cracks in the 3G ONT layer were caused by cyclic loading during the biotribological test. Transverse cracks visible in the wear track image for the 3G ONT layer indicated additional fatigue wear (Figure 11g,h). The analysis of microscopic wear tracks showed that abrasive wear was the dominant mechanism.

### 3.4. Wear Mechanism of Ti–13Zr–13Nb Alloy before and after Anodizing in Ringer’s Solution

**Figure 12:**
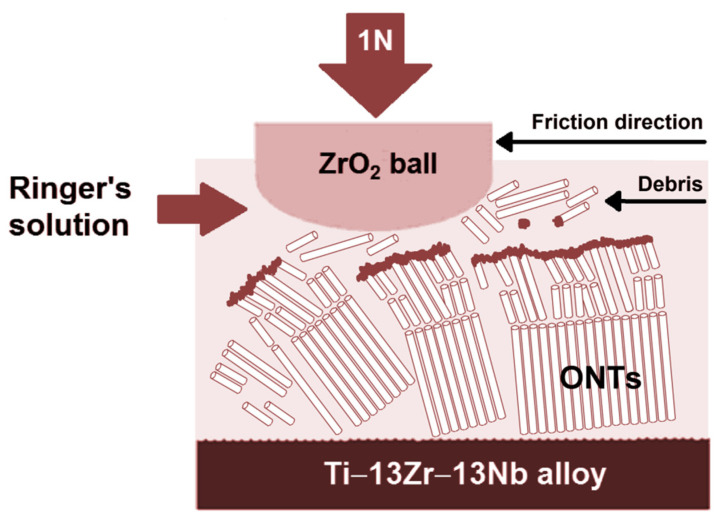
Three-body abrasion wear mechanism of the Ti–13Zr–13Nb alloy before and after anodizing in Ringer’s solution.

The mechanism of biotribological wear of ONT layers on the Ti–13Zr–13Nb alloy substrate in Ringer’s solution is based on the breaking of oxide nanotubes and their densification in the outer part of the oxide layers according to the wear mechanism of three-body abrasive wear [15,16]. In the proposed mechanism, between the surface of the Ti–13Zr–13Nb alloy with a layer of ONTs (body 1) and the surface of the ZrO_2_ ball (body 2), there are particles of worn material (body 3), that act as a carrier abrasive (Figure 12). The wear results from the gradual loss of material in the contact area of the interacting surfaces as body 1 and body 2 move relative to each other.

Wear debris is mainly formed in the form of single or aggregated ONTs as a result of cracking of the oxide layers in various places, which results in the appearance of ONT fragments of various sizes. It was previously observed in the literature that densification of ONTs is accompanied by wear and cracking [41]. The increase in the indentation depth of ONTs causes cracking of oxide nanotubes and bending and cracking of adjacent ONTs resulting in gradual densification of small fragments of ONT layers [47]. As a consequence of the detachment of the ONT layer from the substrate, the remains of the oxide layer are released in the contact area, which can be pushed out of the contact or trapped in it. As soon as sliding starts, it can be expected that wear debris in the contact area will be exposed to mechanical and electrochemical influences, which may occur sequentially or at the same time, contributing to increased wear of the material. It was reported that as a result of continuous smashing and densification of the wear debris in the central area of the wear track, a compact oxide layer is formed [41]. The tribolayer obtained in this way may reveal protective properties against corrosion and biotribological wear of the substrate. Simultaneously with the formation of the tribolayer along with the movement of the counter-sample, part of the wear debris is pushed to the ONT layer surrounding the sliding contact area. This is a probable reason for inducing cyclical compressive stresses, which cause damage to the structure of the surface and subsurface parts of the ONT layer by initiation and propagation of cracks leading to delamination. This revealed that the ONT layers were brittle and had poor adhesion to the Ti–13Zr–13Nb alloy substrate. Xu and co-authors [47] suggested that brittle ONT layers bend elastically to a very small strain and consequently collapse. Through the emerging cracks in the ONT layer, the electrolyte can penetrate into the substrate, which induces electrochemical corrosion and additionally affects the detachment of the oxide layer.

The micromechanical and biotribological properties of the porous ONT layers on the Ti–13Zr–13Nb alloy under wet sliding in Ringer’s solution strongly depend on the anodizing conditions, among which the composition of the electrolyte containing fluoride ions plays a key role.

## 4. Conclusions

The assessment of the effect of anodizing conditions on the micromechanical properties of the Ti–13Zr–13Nb alloy shows that Vickers microhardness determined under variable loads changed depending on the type of electrolyte and applied voltage–time parameters of electrochemical oxidation. Vickers microhardness for the non-anodized alloy was independent of the load used and amounts to 302(1). For 1G, 2G, and 3G ONT layers, the dependence of Vickers microhardness on applied load was revealed due to the differences in the morphological parameters and lengths of the ONTs. For the 1G ONT layer, an increase in Vickers microhardness in the range from 433(1) to 340(7) with increasing load was observed, which was related to the smallest outer diameter of ONTs with the shortest nanotube length. Vickers microhardness decreased from 181(5) to 252(6) and from 254(3) to 221(3) with increasing load for 2G and 3G ONT layers, respectively, compared to the alloy substrate.

Based on the biotribological tests carried out in Ringer’s solution in a reciprocating motion in the ball-on-flat system for the Ti–13Nb–13Zr alloy before and after anodizing, it was found that the non-anodized alloy was characterized by the highest wear resistance for which the average material volume consumption was 8.20(4)·10^−4^ mm^3^ N^−1^ m^−1^. The resistance to abrasive wear decreased for 1G, 2G, and 3G ONT layers, taking the highest value of the average material volume consumption of 1.79(9)·10^−3^ mm^3^ N^−1^ m^−1^ for the 2G ONT layer. It was ascertained that the lower the coefficient of friction, the greater the volumetric wear, i.e., the lower the resistance to abrasive wear. The kinetic coefficient of friction determined based on the friction coefficient, took the smallest value of 0.86(8) for the 2G ONT layer. The highest coefficient of kinetic friction of 0.94(1) was characterized by the surface of the 1G ONT layer. Based on the results obtained, a three-body abrasion wear mechanism was proposed for biotribological wear of the Ti–13Zr–13Nb alloy before and after anodizing in Ringer’s solution.

In this study, the in vitro biotribological properties of the tested biomaterials were studied in protein-free simulated body fluid. In order to create studies under wet sliding more similar to in vivo conditions, future research will focus on the determination of the wettability of ONT layers and biotribology wear assessment in a simulated body fluid with the addition of proteins.

## Figures and Tables

**Table 1:** Conditions for anodic production of 1G [32], 2G [34], and 3G [31] ONTs on the Ti–13Zr–13Nb alloy.

Conditions	ONTs on Ti–13Zr–13Nb		
	I Generation (1G)	II Generation (2G)	III Generation (3G)
Voltage (V)	20	20	50
Time (min)	120	120	80
Electrolyte	0.5% HF	1M (NH_4_)_2_SO_4_ + 2% NH_4_F	1M C_2_H_6_O_2_ + 4% NH_4_F
Inner diameter of ONT (nm)	71(7)	61(11)	218(39)
Outer diameter of ONT (nm)	87(10)	103(16)	362(44)
Length of ONT (μm)	0.94(9)	3.9(2)	9.7(6)

## Data Availability

Not applicable.
